# A document classifier for medicinal chemistry publications trained on the ChEMBL corpus

**DOI:** 10.1186/s13321-014-0040-8

**Published:** 2014-08-12

**Authors:** George Papadatos, Gerard JP van Westen, Samuel Croset, Rita Santos, Simone Trubian, John P Overington

**Affiliations:** European Molecular Biology Laboratory, European Bioinformatics Institute (EMBL-EBI), European Molecular Biology Laboratory, Wellcome Trust Genome Campus, Hinxton, Cambridge CB10 1SD UK

**Keywords:** Machine learning, Triage, Curation, Document classification

## Abstract

**Background:**

The large increase in the number of scientific publications has fuelled a need for semi- and fully automated text mining approaches in order to assist in the triage process, both for individual scientists and also for larger-scale data extraction and curation into public databases. Here, we introduce a document classifier, which is able to successfully distinguish between publications that are `ChEMBL-like’ (i.e. related to small molecule drug discovery and likely to contain quantitative bioactivity data) and those that are not. The unprecedented size of the medicinal chemistry literature collection, coupled with the advantage of manual curation and mapping to chemistry and biology make the ChEMBL corpus a unique resource for text mining.

**Results:**

The method has been implemented as a data protocol/workflow for both Pipeline Pilot (version 8.5) and KNIME (version 2.9) respectively. Both workflows and models are freely available at: ftp://ftp.ebi.ac.uk/pub/databases/chembl/text-mining. These can be readily modified to include additional keyword constraints to further focus searches.

**Conclusions:**

Large-scale machine learning document classification was shown to be very robust and flexible for this particular application, as illustrated in four distinct text-mining-based use cases. The models are readily available on two data workflow platforms, which we believe will allow the majority of the scientific community to apply them to their own data.

**Abstract:**

**Electronic supplementary material:**

The online version of this article (doi:10.1186/s13321-014-0040-8) contains supplementary material, which is available to authorized users.

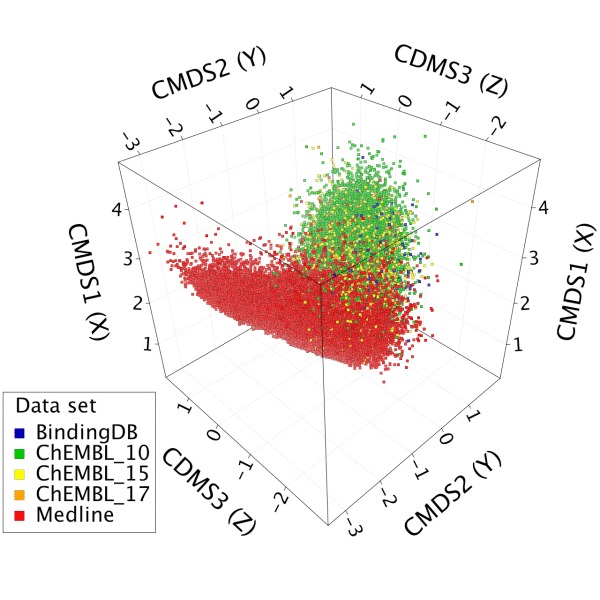

## Background

The ChEMBL database stores a large quantity of 2D compound structures, biological targets, bioactivity data and calculated molecular properties of drugs and drug-like molecules; the coverage of ChEMBL is primarily focused on the medicinal chemistry, chemical biology and drug discovery fields. Data in ChEMBL is manually extracted from experimental results reported in the primary scientific literature and then curated and integrated to ensure consistency and improve data quality [1].

Manual document data entry and curation is expensive and time-consuming [2],[3]. Furthermore, it has become increasingly difficult for curators to keep up with the increasing scientific output produced, and this is likely to become more of an issue as pressure to release more data from funded research programs is applied. Therefore, biomedical researchers, text miners and curators are in need of automated expert systems that can help with the initial steps of the curation process. This phase is known as *triage*, namely the selection of likely relevant scientific articles from large repositories, such as Europe PMC and PubMed [4],[5].

Extracting chemistry-related information from text has been performed in the past, in particular using named entity recognition systems such as Whatizit [6], OSCAR4 [7] or ChemSpot [8]. These tools can help for instance to identify drugs and molecular structures to be further curated or analysed in combination with other data types [9]. However, the main goal of our project diverges from the goal of the tools mentioned. We aim to meet the following criteria: ranking and prioritising the relevant literature using a fast and high performance algorithm, with a generic methodology applicable to other domains and not necessarily related to chemistry and drug discovery. In this regard, we present a method that builds upon the manually collated and curated ChEMBL document corpus, in order to train a Bag-of-Words (BoW) document classifier. The classifier is based on the titles and abstracts of the corpus. The strategy has already proven to be successful in other fields such as toxicogenomics [10],[11], and thus our main aim here has been extension and validation. We demonstrate the use of the methodology and make it available to the community.

In more detail, we have employed two established classification methods, namely Naïve Bayesian (NB) and Random Forest (RF) approaches [12]-[14]. The resulting classification score, henceforth referred to as `ChEMBL-likeness’, is used to prioritise relevant documents for data extraction and curation during the triage process. The data pre-processing workflows and validated models are freely available online under permissive licenses to the community as a Pipeline Pilot protocol and a KNIME workflow respectively [15],[16]. Both the protocol and workflow provide the same functionality and have been validated on the same data set.

## Implementation

**Figure 1:**
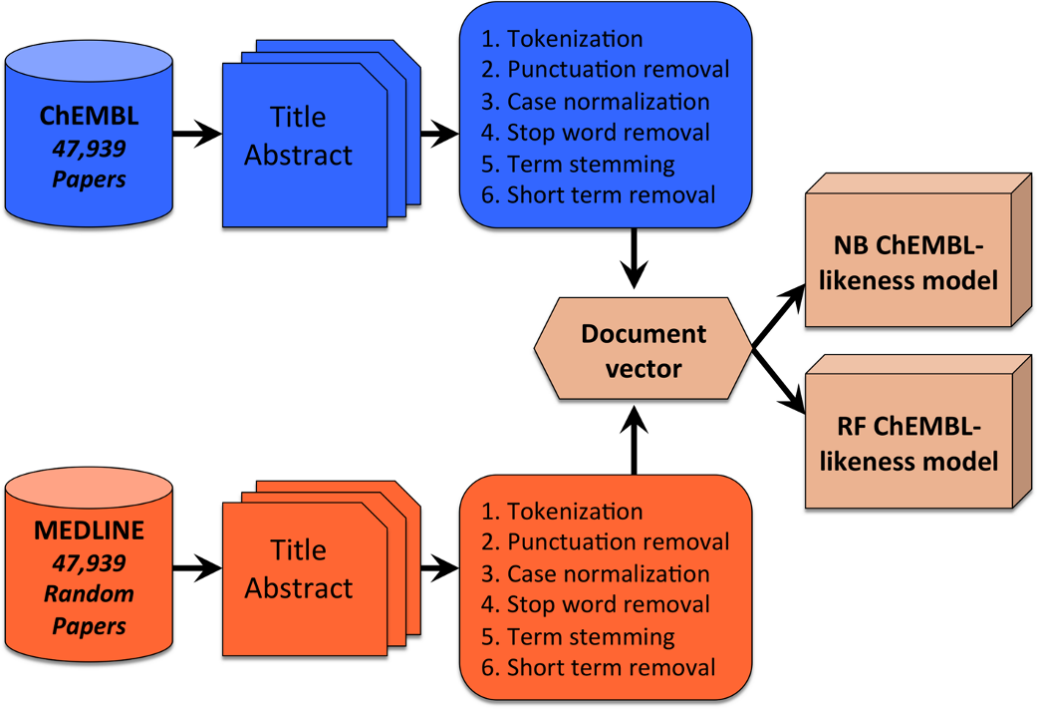
**Document processing and classification workflow.** Abbreviations: NB - Naive Bayesian, RF - Random Forest.

The full set of journal publication titles and abstracts included in ChEMBL (47,939 documents in release 17) was the starting point, while a random but non-overlapping subset of the same size retrieved from MEDLINE [5] was used as the background. The BoW approach was implemented in a standard way by appropriately tokenizing titles and abstracts for the two classes of documents. The resulting terms were submitted to a series of text mining pre-processing operations, such as punctuation removal, case normalisation, removal of stop words (Additional file 1), term stemming and short term removal (<4 characters), see also the example in Table 1. A document vector was then generated for each document, encoding the absence or presence of the remaining terms in a binary string (Table 2). In addition to the single-word document vector, adding word combinations based on n-grams generated a second vector. N-grams were generated by inclusion of pairs (bigrams) and triplets (trigrams) of adjacent words; in these cases stop words or connecting words were kept. The data workflow is schematically summarized in Figure 1.

**Table 1:** **BoW and n-grams example for two document titles**

Source	ChEMBL	Medline
**PubMed ID**	17994679	17886339
**Original title**	Discovery of biaryl anthranilides as full agonists for the high affinity niacin receptor.	Automatic prediction of protein interactions with large scale motion.
**Bag of words**	Discover, biaryl, anthranilid, full, agonist, high, affin, niacin, receptor	Automat, predict, protein, interact, large, scale, motion
**Bigrams**	Dicovery_of, full_agonists, high_affinity, niacin_receptor, …	Automatic_prediction, protein_interaction, large_scale, …
**Trigrams**	Discovery_of_biaryl, high_affinity_niacin, affinity_niacin_receptor, …	Automatic_prediction_of, protein_interaction_with, large_scale_motion, …

**Table 2:** **A document vector example from the titles of the documents in Table** 1

PubMed ID	Discover	Biaryl	Niacin	Receptor	Automat	Predict	Large	…
**17994679**	1	1	1	1	0	0	0	…
**17886339**	0	0	0	0	1	1	1	…

**Figure 2:**
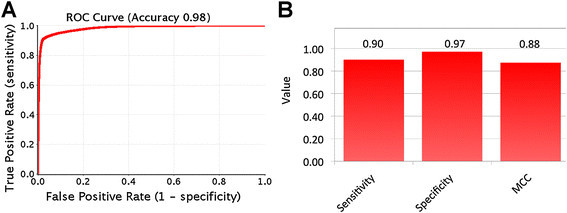
**Receiver operator characteristic curve and external validation performance (Pipeline Pilot model).** The ROC curve generated by a Bayesian classifier (`Learn Good From Bad’ component) in the 80% - 20% stratified partition validation is shown in **(A)**. The performance of this classifier in the test set is shown in **(B)**. Abbreviations: Matthews Correlation Coefficient – MCC, Receiver Operator Characteristic – ROC.

**Figure 3:**
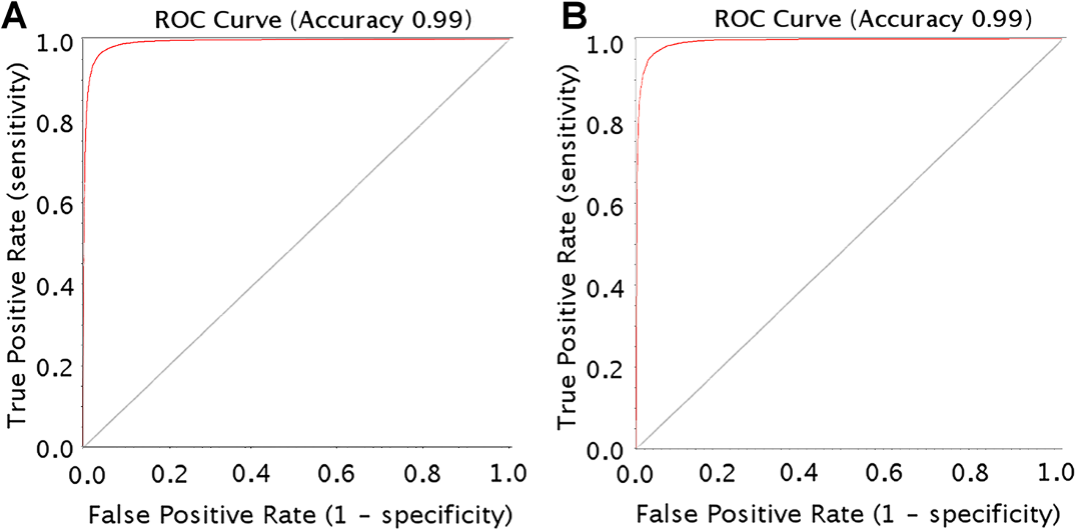
**ROC curve and external validation performance (KNIME model).** The ROC curves were generated by a Random Forest model (`Tree Ensemble Learner’ node). Plot **A** shows the ROC curve for the out-of-bag classification. Plot **B** shows the ROC curve for the 80% - 20% stratified cross validation.

The vectors were then used as binary descriptors for NB and RF classifiers on Pipeline Pilot and KNIME respectively (Table 3). The classifiers were validated in three distinct ways. Firstly by 80%-20% stratified external-validation, see Figures 2 and 3, respectively. The parameters used for the two respective classifiers are described in the Additional file 2. Secondly, the PP classifier was trained on ChEMBL release 10 and prospectively validated on new, unseen publications added in release 17. Finally, the PP classifier was validated on novel and relevant (articles not present in ChEMBL), positive examples that were retrieved from BindingDB [17] – a database of similar scope to ChEMBL.

**Table 3:** **Summary of classification validation statistics across different methods and validation sets**

Method/validation set	AUC	MCC	Sensitivity	Specificity
NB EV	0.98	0.88	0.90	0.97
NB n-grams EV	1.00	0.91	0.95	0.96
NB ChEMBL_17	0.96	0.90	0.92	0.98
NB BindingDB	0.97	0.79	0.80	0.97
RF EV	0.99	0.92	0.95	0.97
RF CV Out-of-Bag	0.99	0.92	0.94	0.97

*Abbreviations: AUC* Area Under the Curve, *CV* cross validation, *EV* external validation, *MCC* Matthews Correlation Coefficient, *NB* Naive Bayesian, *RF* Random Forest.

What is noteworthy from the ROC curves in Figures 2 and 3 is that the classifiers appear to have a very high true positive rate at the start of the curve. To quantify this we ranked the predictions in the external validation by the model value rather than class. In the top 5% only 4 out of 954 are false positives, the remaining 950 are true positives. Likewise in the top 10%, 16 out of 1908 are false positives with 1892 true positives. This could indicate that the classifier is able to accurately rank the documents, i.e. highly ranked documents indicate more desirable papers. Currently we are validating this observation (see section “Filtering allosteric ligand-related publications”).

Using the n-gram based document vector was found to slightly improve performance during the stratified partition validation at the expense of an increase in training time and resource usage (3 minutes to completion for BoW and 9 minutes to completion for n-grams on the same machine with the same data, increase of approximately 300%), while performance only increased by 2.5% on average. Given the minimal increase in predictive performance, it was chosen not to follow this up with the other validation strategies. However, it might be interesting to try this approach on sets where the BoW method performs inadequately as we did observe an improvement. Overall, the positive retrospective and prospective validation statistics indicate that these models are suitable to identify highly relevant articles for subsequent information extraction.

### Classification validation parameters

Performance of the classifier was estimated based on Sensitivity, Specificity, and Matthews Correlation Coefficient (MCC). Sensitivity is the fraction of true positive predictions of the total positive (ChEMBL-like) documents: True Positives / (True Positives + False Negatives). Similarly, specificity is the fraction of true negative predictions of the total negative (non ChEMBL-like) documents: True Negatives / (True Negatives + False Positives). Finally the Matthews correlation coefficient is calculated as follows:

1 $$\mathrm{MCC}=\frac{\left(\mathrm{TP}*\mathrm{TN}\right)-\left(\mathrm{FP}*\mathrm{FN}\right)}{\sqrt{\left(\mathrm{TP}+\mathrm{FP}\right)*\left(\mathrm{TP}+\mathrm{FN}\right)*\left(\mathrm{TN}+\mathrm{FP}\right)*\left(\mathrm{TN}+\mathrm{FN}\right)}}$$

**Figure 4:**
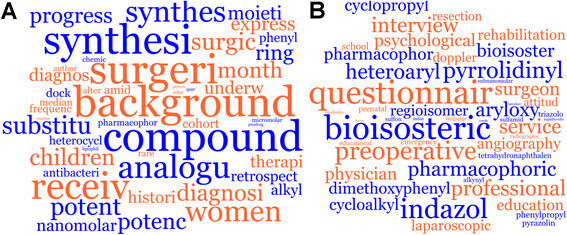
**Word cloud visualization of feature importance according to the NB model (A) and RF model (B).** More important terms are depicted in larger and bolder type. Blue coloured terms are correlated with ChEMBL whereas orange ones are correlated with the MEDLINE class.

In addition to the performance statistics across a number of validation sets, we also looked at the relative importance of the terms/features according to the two models. In order to assess the importance for each individual feature, we employed (i) the Bayesian score derived by the NB model and ii) how frequently a feature was used for a split in the first three levels of a tree, across all the trees of the Random Forest model. The first level of the tree, in particular, correlates with the Gini Importance metric [14]. Figure 4 depicts the 48 most important features/words in terms of their importance for both the NB and the RF model. As expected, terms such as *compound* and derivatives of *potency*, *analogue* and *synthesis* are among the most important with the highest discriminative power for the model. In addition, terms that are unlikely to occur in ChEMBL-like publications, such as *psychological, surgery*/*surgical* and *children* are also listed as important.

## Results and discussion

Four applications and use cases that leverage the classifier functionality are presented below. Two applications rely on the quantification of the ChEMBL-likeness score, one application is focused on a specific disease area, and finally a fourth application aims at identifying papers that are relevant to a less-defined, more complex concept (age-related differential drug response).

### Prioritizing new publications

**Figure 5:**
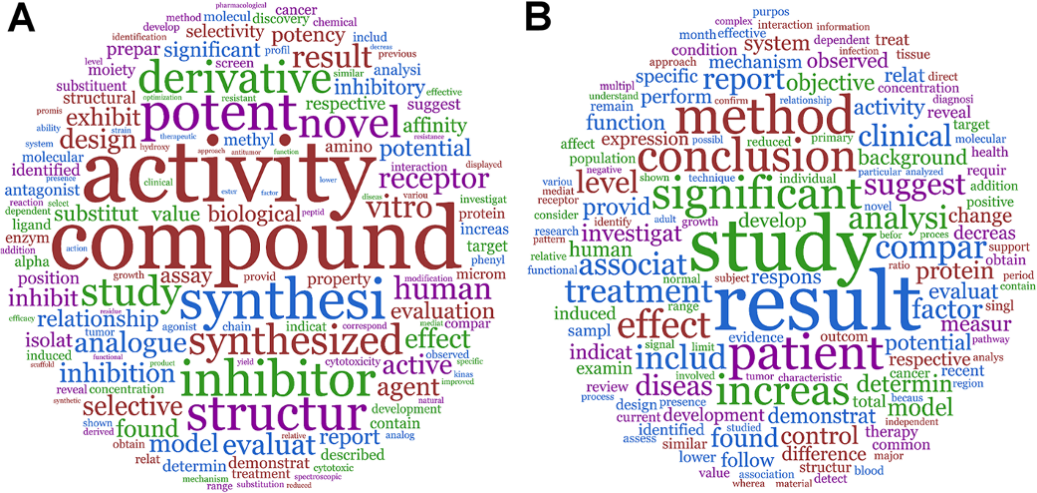
**Word cloud visualization of the ChEMBL and MEDLINE data sets. (A)** Words most frequent in the ChEMBL corpus (more frequent words are depicted larger). A large emphasis on chemistry related terms is apparent. **(B)** Word cloud visualization of the words most frequent in our MEDLINE background set. Here an emphasis on clinical data can be observed.

The main application of the method is the automated scoring and prioritization of new papers according to their relevance to the ChEMBL corpus. Summary level, word differences between medicinal chemistry literature and the MEDLINE corpus are visualized in Figure 5. Note that the raw frequencies are visualized here, contrary to Figure 4, which shows the terms deemed to be most important according to the models. Interestingly, terms such as *compound*, *potent*, and *synthesized* appear in both, indicating that they are common and important words. The classifier will be used to identify interesting papers to be included in ChEMBL in journals not routinely covered (and thus at the moment potentially missed).

### Filtering allosteric ligand-related publications

**Figure 6:**
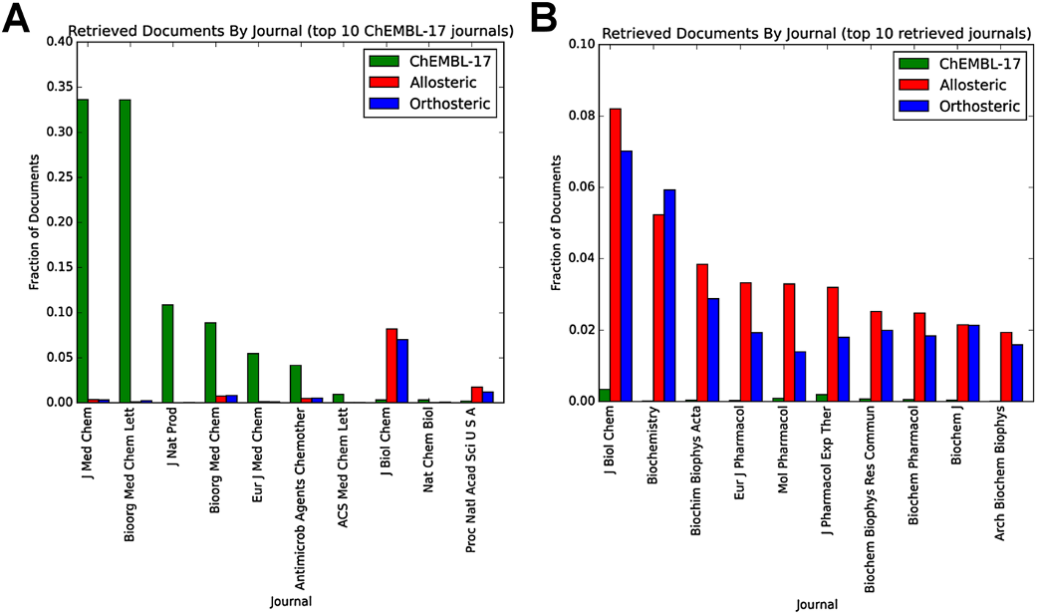
**Complementarity to current literature in ChEMBL.** Several medicinal chemistry journals are routinely covered in ChEMBL **(A)**. The ChEMBL-likeness classifier is able to retrieve relevant papers from journals that are not routinely covered **(B)**.

A second use case concerned analysis of publications on allosteric modulators. Following up on previous work [18], a large corpus of publications was retrieved containing one or more keywords related to allosteric modulators (Additional file 3). The ChEMBL-likeness score derived from the classifier was employed to filter the total number of retrieved documents and prioritise only relevant publications on medicinal chemistry. This approach reduced the initial set of documents from 60,924 to 12,730, a far more manageable number for subsequent expert analysis and filtering. Additionally, when an average cost per article of $30 is assumed based on pay-per-view access [19], this corresponds to a cost reduction of $1,445,820 for this subset alone. In practice the cost reduction might not be as high as sketched here. A human curator is equally able to differentiate between relevant and non-relevant papers based on the title and abstract. However, a curator is still paid for this task and selecting articles would prevent them from reading full texts and curating data. Hence, reducing the time required for selection by removing a large irrelevant fraction should lead to direct increases in efficiency (i.e. the amount of data points to be added to a resource such as ChEMBL per dollar). In this use case the bag of words classifier demonstrated that it was able to pick up papers in journals that are underrepresented in ChEMBL and hence that the classifier was able to retrieve papers complementary to the ChEMBL corpus (Figure 6).

While a detailed analysis of this set will be reported elsewhere we would like to outline our approach for validating the ability of our models to rank papers. Initially we have looked at several samples from the set and indeed higher scoring documents appear to be more relevant whereas low scoring documents that are still ChEMBL-like contain relatively more false positives. Some examples are PMID:11142631 (highest scoring ChEMBL-like), PMID:9891064 (lowest scoring ChEMBL-like), PMID:17008604 (non-ChEMBL-like). To further validate the ranking ability, we have selected a top 10 (based on classifier score) per journal of documents that are predicted to be ChEMBL-like. After these documents have been curated we will compare the score and relevance for ChEMBL. As we have gathered these documents from diverse journals this can likely tell us more about the models’ ability to rank documents.

### Generating antimalarial paper alerts

**Figure 7:**
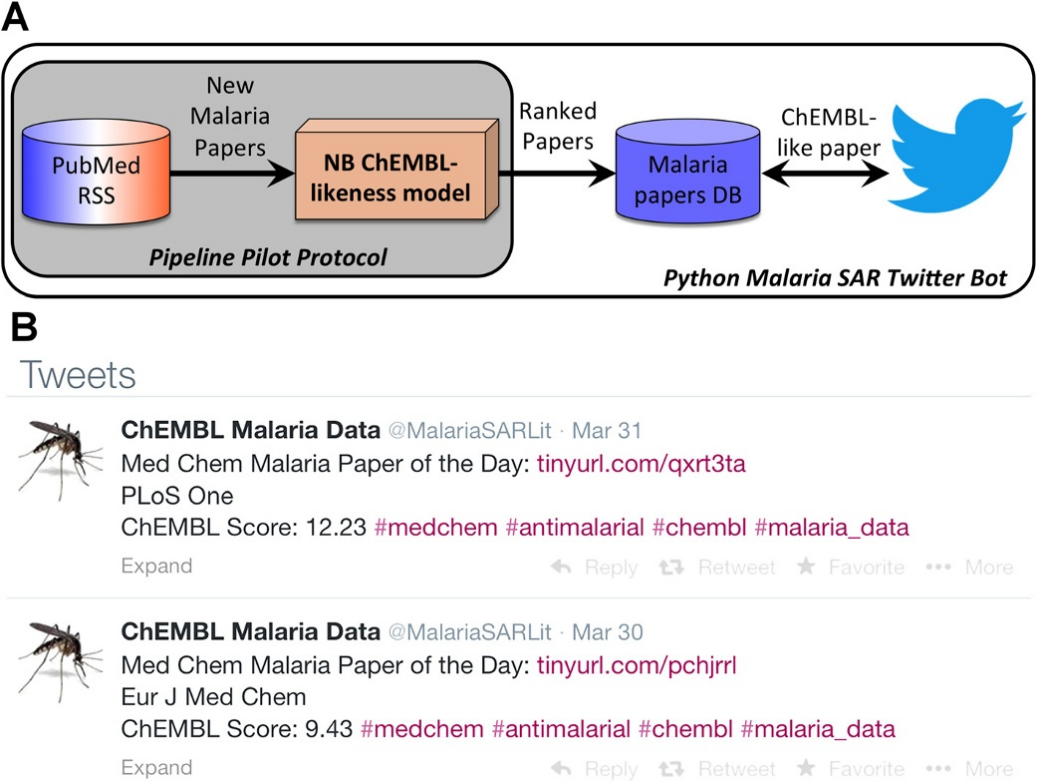
**The @MalariaSARLit twitter bot.** Schematic overview of the pipeline, controlled by an automated Python script **(A)**. Examples of daily tweets with alerts for recent medicinal chemistry anti-malarial publications **(B)**. The latter are automatically prioritized using the NB document classification model.

In an effort to share the utility of the classifier with the scientific community, a Twitter account was set up (@MalariaSARLit) which tweets malaria medicinal chemistry publications on a daily schedule. The account is controlled by an automated Python script (effectively a Twitter bot) which: (i) monitors the PubMed RSS daily for new malaria-related publications, i.e. publications containing the keyword `malaria’ in either their title or abstract; (ii) scores them according to the ChEMBL-likeness NB document classification score; (iii) stores the results in a relational database table for further analysis; and finally (iv) tweets a randomly selected ChEMBL-like publication at 1 pm GMT every day (Figure 7). The twitter feed is also displayed as a widget at the Home page of the Malaria-Data resource provided by the EMBL-EBI [20]. It would be trivial to apply the same methodology for other disease terms retrospectively or prospectively and produce a repository of prioritized relevant publications for further curation and annotation.

### Identifying age-related differential drug responses

The method can be easily adapted to a more complex task, namely the retrieval and prioritization of articles where age-related differential drug responses are reported. After filtering out articles not containing age- and drug- related words based on a dictionary, the NB classifier was trained and validated on manually checked publication abstracts. The articles selected to train and validate the NB classifier contained at least 5 age- and/or drug-related words (Additional file 4). A “relevant” flag was assigned if the abstract contained pertinent information about drugs with reported age-related differential drug responses. Similarly a “non-relevant” flag was attributed if the information was deemed irrelevant. For a fair representation, an equal number of articles from both sets were used to train (in total 125 articles) and validate the model. In the end, the model scored the likelihood of an article to contain information about drugs that are not as effective or safe in paediatric or geriatric populations when compared with adult populations. This approach identified and prioritized approximately 1,400 articles out of a pool of 19,200. Articles freely available in PubMed central were selected for further evaluation. From the 168 selected, 19 contained relevant information, resulting in the identification of 46 new drugs with reported age-related differential drug responses. Despite its apparently modest performance, the classifier has highlighted articles, which had not been previously identified by conventional literature search methods, hence contributing considerably to expand the current list of drugs with known age-related response differences.

## Conclusions

In conclusion, this method provides a fast and robust way to automatically identify and score articles relevant to the medicinal chemistry, chemical biology and drug discovery fields. The versatility of the method is highlighted here with four distinct applications, although there are many more that could be foreseen. Both PP (NB) and KNIME (RF) workflows and models, along with the PubMed identifiers of the documents used in training and test sets respectively, are available on the ChEMBL ftp server. This will ensure the reproducibility and reuse of our methodology and the straightforward dissemination of the models *via* two popular and user-friendly workflow platforms.

While it could be possible that usage of full text or named entity recognition increases performance over the usage of abstracts and titles alone, there is in reality little room for improvement, as shown in the models trained on n-grams as opposed to BoW data. This equally true for the inclusion of other sources of information like author names or journal name and for the investigation into potential data fusion methods relying on both RF and NB. However, another potential result can be that inclusion of this data actually limits the broad applicability of the classifier. These and other potential improvements are the subject of further on-going studies. We propose that titles and abstracts alone, as opposed to full text or annotated documents, provide sufficient information content for a reliable initial classification on a large scale avoiding unrequired complexity as is required in our use cases.

Notably, the way in which the contents of documents are abstracted here bears similarities to established chemoinformatics techniques. The document vector (presence or absence of words drawn from a dictionary) is obviously analogous to a dictionary-based fingerprint, whereby the dictionary is not predefined but constructed from the underlying data. In the same sense, word tokens are analogous to a compound’s substructural features while the word n-grams are linear combination of features (word tokens), which are in turn similar to the substructural features extracted from path-based fingerprints. As a result, this allows for the introduction of additional approaches from the chemoinformatics domain to text mining, including, but not limited to, document clustering, applicability domain determination for classification models, as well as feature importance determination (although this was touched upon already above). Finally, we aim to expand the scope of this model by applying it to chemical patent document mining in the near future. Here, we could score and prioritise relevant patent documents based on the title and abstract content.

## Availability and requirements

**Project name:** ChEMBL literature classifier - Pipeline Pilot and KNIME workflows

**Project home page:**ftp://ftp.ebi.ac.uk/pub/databases/chembl/text-mining, and https://github.com/chembl/chembl_literature_classifier

**Operating system(s):** OS X and Windows

**Programming language:** Java/Pilot Script

**Other requirements:** KNIME (version 2.9) or Pipeline Pilot (version 8.5) installed

**License:** Apache 2 License

**Any restrictions to use by non-academics:** None

## Authors’ contributions

GP, GvW, and JPO conceived the study/method. GvW, GP, ST, and RS participated in its design and application. GP, GvW, SC, RS, ST, and JPO helped drafting the manuscript. All authors read and approved the final manuscript.

## Additional files

## Electronic supplementary material

Additional file 1: Is a list of stop words used.(PDF 40 KB)

Additional file 2: Describes the classifier parameters.(PDF 51 KB)

Additional file 3: Is a list of allosteric-words.(PDF 30 KB)

Additional file 4: Is a list of age- and drug-related words.(PDF 30 KB)

Below are the links to the authors’ original submitted files for images.

Authors’ original file for figure 1

Authors’ original file for figure 2

Authors’ original file for figure 3

Authors’ original file for figure 4

Authors’ original file for figure 5

Authors’ original file for figure 6

Authors’ original file for figure 7

Authors’ original file for figure 8

## Abbreviations

AUC: Area under the curve
CV: Cross validation
EV: External validation
MCC: Matthews correlation coefficient
NB: Naive Bayesian
RF: Random forest
Sens: Sensitivity
Spec: Specificity
