# Supplementary material for: A document classifier for medicinal chemistry publications trained on the ChEMBL corpus
Source: J Cheminform. 2014 Aug 12;6:40. doi: 10.1186/s13321-014-0040-8 (PMC4158272; doi:10.1186/s13321-014-0040-8)
Supplement: Supplementary file 1 — Additional file 1: Is a list of stop words used.(PDF 40 KB) [file 13321_2014_40_MOESM1_ESM.pdf]

Additional File 1 - Classifier parameters

Learn Good From Bad Pipeline Pilot component - Naive Bayesian classifier

The NB classifier employs a probabilistic model based on the simplifying assumption of conditional independence among features. Although this assumption is rarely true in real life, the performance of NB has been empirically found to be comparable to other more sophisticated machine learning methods across a number of diverse domains, including text mining applications. The PP component builds a two-class Laplacian-modified Bayesian classification model. Here, the model distinguishes 'ChEMBL-like' documents from non 'ChEMBL-like' ones, based on the frequency of occurrence and distribution of their terms across the two classes.

The default parameters were used for the Pipeline Pilot component and in Learn Options the option Validate Models was chosen.

Tree Ensemble Learner KNIME node - Random Forest classifier

An RF is an ensemble of unpruned decision trees, where each tree is built with a random subset of the data and at each node the most important feature is chosen from a random subset of features. According to the Tree Ensemble KNIME node, each of the decision tree models is learned on a different set of rows (data records), such as documents and a different set of columns (attributes), such as the elements of a document vector. The output model describes an ensemble of decision tree models and is generating predictions using the majority vote.

The configuration parameters used in the KNIME node generate a model that is similar to the Random Forest classifier:

Split Criterion: Gini index
Number of levels (tree depth): unlimited
Minimum node size: unlimited
Number of models: 750
Data Sampling: Use a fraction of 0.8 and sample with replacement (bootstrapping)
Attribute Sampling: Sample using the square root of the number of attributes for each tree node split
