# Supplementary material for: A document classifier for medicinal chemistry publications trained on the ChEMBL corpus
Source: J Cheminform. 2014 Aug 12;6:40. doi: 10.1186/s13321-014-0040-8 (PMC4158272; doi:10.1186/s13321-014-0040-8)
Supplement: Supplementary file 2 — Additional file 2: Describes the classifier parameters.(PDF 51 KB) [file 13321_2014_40_MOESM2_ESM.pdf]

Additional File 2 – Stop words used

1
2
3
4
5
6
7
8
9
a
about
above
after
again
against
all
am
an
and
any
are
aren't
as
at
be
because
been
before
being
below
between
both
but
by
can't
cannot
could
couldn't
did
didn't
do
does
doesn't
doing
don't
down
during

each
few
for
from
further
had
hadn't
has
hasn't
have
haven't
having
he
he'd
he'll
he's
her
here
here's
hers
herself
him
himself
his
how
how's
i
i'd
i'll
i'm
i've
if
in
into
is
isn't
it
it's
its
itself
let's
me
more
most
mustn't
my
myself
no
nor

not
of
off
on
once
only
or
other
ought
our
ours
ourselves
out
over
own
same
shan't
she
she'd
she'll
she's
should
shouldn't
so
some
such
than
that
that's
the
their
theirs
them
themselves
then
there
there's
these
they
they'd
they'll
they're
they've
this
those
through
to
too
under

until
up
very
was
wasn't
we
we'd
we'll
we're
we've
were
weren't
what
what's
when
when's
where
where's
which
while
who
who's
whom
why
why's
with
won't
would
wouldn't
you
you'd
you'll
you're
you've
your
yours
yourself
yourselves
across
almost
alone
along
already
also
although
always
among
another
anybody

anyone
anything
anywhere
area
areas
around
ask
asked
asking
asks
away
b
back
backed
backing
backs
became
become
becomes
began
behind
beings
best
better
big
c
came
can
case
cases
certain
certainly
clear
clearly
come
d
differ
different
differently
done
downed
downing
downs
e
early
either
end
ended
ending

ends
enough
even
evenly
ever
every
everybody
everyone
everything
everywhere
f
face
faces
fact
facts
far
felt
find
finds
first
four
full
fully
furthered
furthering
furthers
g
gave
general
generally
get
gets
give
given
gives
go
going
good
goods
got
great
greater
greatest
group
grouped
grouping
groups
h
high

higher
highest
however
important
interest
interested
interesting
interests
j
just
k
keep
keeps
kind
knew
know
known
knows
l
large
largely
last
later
latest
least
less
let
lets
like
likely
long
longer
longest
m
made
make
making
man
many
may
member
members
men
might
mostly
mr
mrs
much
must

n
necessary
need
needed
needing
needs
never
new
newer
newest
next
nobody
non
noone
nothing
now
nowhere
number
numbers
o
often
old
older
oldest
one
open
opened
opening
opens
order
ordered
ordering
orders
others
p
part
parted
parting
parts
per
perhaps
place
places
point
pointed
pointing
points
possible
present

presented
presenting
presents
problem
problems
put
puts
q
quite
r
rather
really
right
room
rooms
s
said
saw
say
says
second
seconds
see
seem
seemed
seeming
seems
sees
several
shall
show
showed
showing
shows
side
sides
since
small
smaller
smallest
somebody
someone
something
somewhere
state
states
still
sure
t

take
taken
therefore
thing
things
think
thinks
though
thought
thoughts
three
thus
today
together
took
toward
turn
turned
turning
turns
two
u
upon
us
use
used
uses
v
w
want
wanted
wanting
wants
way
ways
well
wells
went
whether
whole
whose
will
within
without
work
worked
working
works
x

y
year
years
yet
young
younger
youngest
z
