# Supplementary material for: A document classifier for medicinal chemistry publications trained on the ChEMBL corpus
Source: J Cheminform. 2014 Aug 12;6:40. doi: 10.1186/s13321-014-0040-8 (PMC4158272; doi:10.1186/s13321-014-0040-8)
Supplement: Supplementary file 3 — Additional file 3: Is a list of allosteric-words.(PDF 30 KB) [file 13321_2014_40_MOESM3_ESM.pdf]

Additional File 3 – Allosteric words used

uncompetitive
Uncompetitive
un-competitive
Un-competitive
non-competitive
Non-competitive
noncompetitive
Noncompetitive
allostery
Allostery
alostery
Alostery
activators
Activators
positive modulators
negative modulators
regulatory site
Regulatory site
positive modulator
negative modulator
secondary binding site
Secondary binding site
secondary pocket
Secondary pocket
nonsubstrate
Nonsubstrate
allosteric
Allosteric
allosterism
Allosterism
alosteric
alosterism
Alosteric
Alosterism
indirectly inhibit
indirectly activate
NNRTI
non-nucleoside reverse transcriptase inhibitor
