# Supplementary material for: A document classifier for medicinal chemistry publications trained on the ChEMBL corpus
Source: J Cheminform. 2014 Aug 12;6:40. doi: 10.1186/s13321-014-0040-8 (PMC4158272; doi:10.1186/s13321-014-0040-8)
Supplement: Supplementary file 4 — Additional file 4: Is a list of age- and drug-related words.(PDF 30 KB) [file 13321_2014_40_MOESM4_ESM.pdf]

Additional File 4 – Age related words used

adolescence
adolescent
adolescents
adolescents versus children
adult
adults
adults versus children
adults versus teenagers
age
age group
age groups
babies
baby
child
childhood
children
children versus adolescent
children versus adolescents
children versus adult
children versus adults
children versus elderly
children versus teenager
children versus teenagers
differential
dose
dosing
effect
effective
elderly
elderly versus adults
elderly versus children
geriatric
geriatric population
infant
infants
juvenille
kid
kids
menarche
neonate
neonates
newborn
newborns
peadiatric
peadiatric populations
pediatric
pediatric population

pediatric versus adult
puberty
response
safe
teenager
teenagers
teenagers versus adults
teenagers versus children
young
