# Supplementary figures and images for: A document classifier for medicinal chemistry publications trained on the ChEMBL corpus
Source: J Cheminform. 2014 Aug 12;6:40. doi: 10.1186/s13321-014-0040-8 (PMC4158272; doi:10.1186/s13321-014-0040-8)

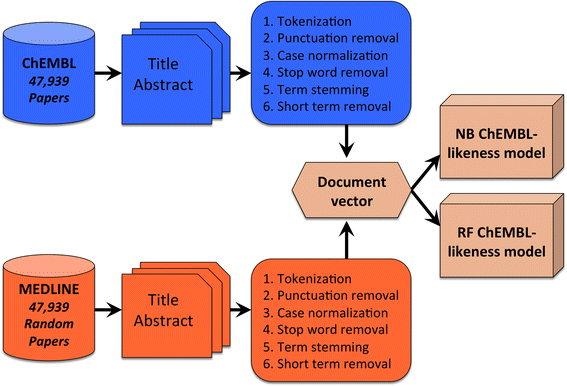

Supplement: Supplementary file 5 — Authors’ original file for figure 1 [file 13321_2014_40_MOESM5_ESM.gif]

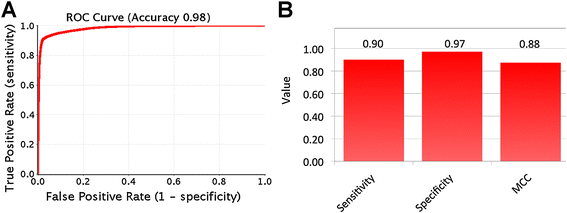

Supplement: Supplementary file 6 — Authors’ original file for figure 2 [file 13321_2014_40_MOESM6_ESM.gif]

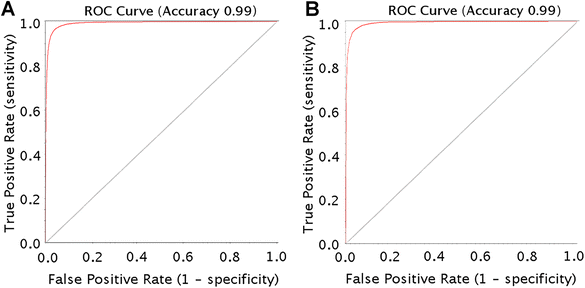

Supplement: Supplementary file 7 — Authors’ original file for figure 3 [file 13321_2014_40_MOESM7_ESM.gif]

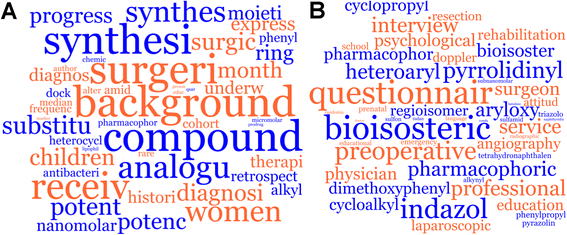

Supplement: Supplementary file 8 — Authors’ original file for figure 4 [file 13321_2014_40_MOESM8_ESM.gif]

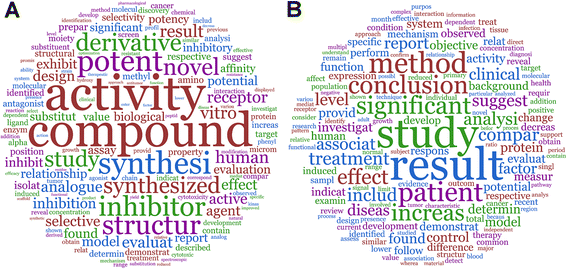

Supplement: Supplementary file 9 — Authors’ original file for figure 5 [file 13321_2014_40_MOESM9_ESM.gif]

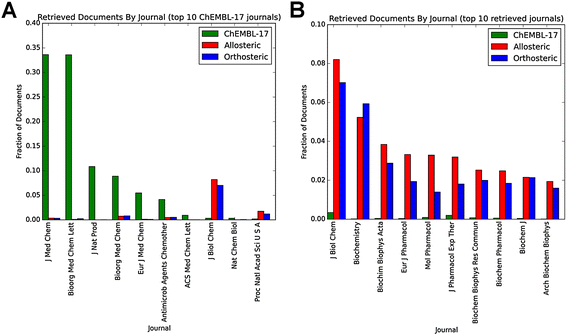

Supplement: Supplementary file 10 — Authors’ original file for figure 6 [file 13321_2014_40_MOESM10_ESM.gif]

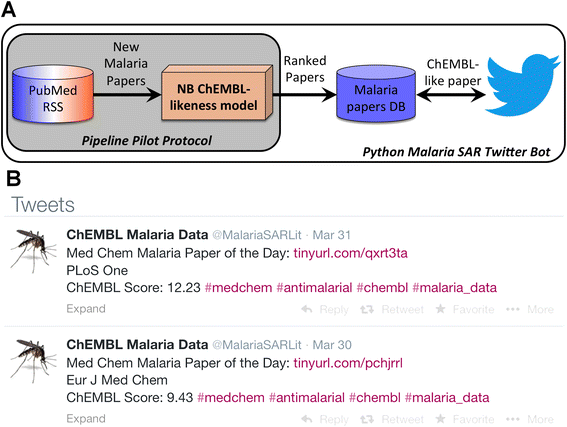

Supplement: Supplementary file 11 — Authors’ original file for figure 7 [file 13321_2014_40_MOESM11_ESM.gif]
